# Optic nerve sheath fenestration for visual impairment in cerebral venous diseases

**DOI:** 10.3389/fneur.2023.1065315

**Published:** 2023-01-24

**Authors:** Xiao Xue, Chen Zhou, Yuan Gao, Xunming Ji, Xuxiang Zhang

**Affiliations:** ^1^Department of Ophthalmology, Xuanwu Hospital, Capital Medical University, Beijing, China; ^2^Department of Neurology, Xuanwu Hospital, Capital Medical University, Beijing, China; ^3^Laboratory of Brain Disorders, Ministry of Science and Technology, Beijing Institute of Brain Disorders of Capital Medical University, Beijing, China; ^4^Department of Biomedical Engineering, School of Biological Science and Medical Engineering, Beihang University, Beijing, China; ^5^Department of Neurosurgery, Xuanwu Hospital, Capital Medical University, Beijing, China; ^6^Department of Ophthalmology, Beijing Tiantan Hospital, Capital Medical University, Beijing, China

**Keywords:** optic nerve sheath fenestration, papilledema, cerebral venous diseases, cerebral venous stenosis, cerebral venous thrombosis

## Abstract

**Objective:**

Visual impairment is the most common clinical feature of cerebral venous sinus occlusion or cerebral venous thrombosis-induced intracranial hypertension, which can result in optic atrophy, leading to irreversible vision loss, visual field defections, and finally, permanent blindness. Papilledema is a typical early pathophysiological alteration in visual impairment. Optic nerve sheath fenestration (ONSF) has become increasingly accepted as an option to prevent or halt progressive visual loss owing to its low risk and complications. The objective of this study is to review the latest research progress on ONSF for the treatment of visual impairment related to cerebral venous diseases.

**Methods:**

Study were searched following PRISMA guidelines based on three electronic databases (Pubmed, Embase and Medline-Ovid). We used the following keywords and variations as keywords to identify studies: “optic nerve sheath fenestration, papilledema, cerebral venous diseases, cerebral venous stenosis, cerebral venous thrombosis, idiopathic intracranial hypertension”. The publication date of studies was restricted between 1,872.1.1 and 2,021.12.31. The application of ONSF in papilledema due to cerebral venous diseases is reviewed. Additionally, the common surgical approaches as well as advantages and disadvantages are also described graphically.

**Results:**

With the improvement of specific details of the ONSF procedure and surgical instruments, complications of ONSF have reduced and its safety has been significantly improved, although the number of clinically investigated cases in the literature remains low.

**Conclusion:**

We recommend that ONSF should be considered as an imperative alternative to reduce or delay the visual morbidity of cerebral venous diseases, although there is yet no consensus on the optimal surgical timing.

## 1. Introduction

The cerebral venous circulation consists of a superficial venous system and a deep venous system, both of which eventually drain into the right atrium *via* the internal jugular vein and the sigmoid sinus. Proper functioning of the brain relies on coordination of the functions of the arterial and cerebral venous systems. A disturbance in the cerebral venous return can lead to a wide range of neuropathological insults, including cranial hypertension, blood-brain barrier breakdown, abnormal cerebrospinal fluid circulation, impaired glymphatic system function, and hemodynamic disturbances. Causes of cerebral venous reflux disorders can be classified as thrombotic and non-thrombotic: thrombotic disorders include cerebral venous thrombosis (CVT), whereas non-thrombotic disorders include cerebral venous sinus stenosis (CVSS).

## 2. Cerebral venous thrombosis

CVT is a specific type of cerebrovascular disease characterized by disturbed cerebral venous return and cranial hypertension, which presents clinically as headache, visual impairment, seizures, and focal neurological dysfunction (1, 2). CVT most often occurs in young women aged 25–35 years, accounting for ~ 0.5% of all deaths. CVT-related deaths include dural sinus thrombosis and cortical venous thrombosis, in which dural sinus thrombosis accounts for the majority. Venous thrombosis often originates from sinus thrombosis; venous thrombosis alone is rare. Various genetic or secondary thrombogenic factors can be the etiology of cerebral venous stenosis, including Factor V Leiden mutation; thrombin G20210A mutation; hyperhomocysteinemia; protein S, protein C, or antithrombin III defect; pregnancy; puerperium; oral contraceptives; obesity; blood disease; infection; trauma; tumor; dehydration; connective tissue disease; nephrotic syndrome; neurosurgery; and COVID-19 related etiology (2–8).

The clinical manifestations of cerebral venous sinus thrombosis (CVST) are complex and heterogeneous. Blockage of the venous sinus by thrombotic tissue leads to an increase in pressure and venous dilatation in the distal sinus and at the same time, a decrease in cerebral perfusion pressure. These pathological changes can lead to insufficient local perfusion of brain tissue, tissue hypoxia, and reduced cerebrospinal fluid (CSF) absorption, and finally, venous infarction and high intracranial pressure (9, 10). In addition, thrombosis can damage the vascular wall, leading to blood-brain-barrier destruction, vascular rupture, and even subarachnoid hemorrhage. These pathological changes can cause headache, visual impairment, focal nerve injury, seizures, high intracranial pressure, and other symptoms or signs. The VENOST study (11) reported the clinical characteristics of 1,144 CVST patients, of which, 47% were acute onset, 34% were subacute onset, and 19% were chronic onset. The clinical symptoms were headache (87%), nausea and vomiting (28%), visual field defects (27%), seizures (24%), other focal neurological deficits (18%), and consciousness disturbance (18%). In the ISCVST study, papilledema was present in the acute phase in 30% of patients, and 6.7% had a major problem with visual impairment during their visit (12). In the VENOPORT study, 13% of the patients had impaired vision and 1.4% had severe visual loss (13). Moreover, 27% of CVST patients in the ENOST study had blurry vision (14).

## 3. Cerebral venous sinus stenosis

Cerebral venous stenosis is a cerebrovascular disease characterized by disturbed cerebral venous flow return. Based on the location of the lesion, it can be divided into cerebral venous sinus stenosis (CVSS) and internal jugular vein stenosis (IJVS). The mechanism of the generation of CVSS is as follows: (1) Endotype: malformation of the venous sinus wall (arachnoid granulation, scar tissue). The emergence of anatomical variations of the venous sinus wall (venous partition and blind pouch structures), result in a decline in venous compliance and capacity. Stagnation of blood flow or an excessively fast flow rate can thus be produced, eventually leading to an increase in the intravenous pressure and subsequently to an increase in intracranial pressure; (2) Exogenous: is mostly found in patients with self-limited venous sinus collapse caused by increased CSF pressure and extrusion of the intracranial space; however, the etiology remains unknown (15).

Obstruction of venous return by venous sinus stenosis causes increased venous sinus pressure distal to the stenosis, which in turn results in malabsorption of the CSF and increased intracranial pressure. Magnetic resonance imaging identified venous sinus stenosis is up to 93% of patients with idiopathic cranial hypertension (IIH) (14). However, it is worth noting that some patients who have a long-standing variant of venous sinus stenosis may not have an obvious clinical manifestation. Significant intracranial pressure elevations must develop after a “second hit”, which is mostly occurs in cases that are overweight or have CSF hypersecretion (16). Not only is intracranial venous sinus stenosis etiologically complex, but its causal relationship with raised intracranial pressure remains ambiguous. Usually, however, the clinical manifestations of intracranial venous sinus stenosis are associated with increased intracranial pressure, such as headache, pulsatile tinnitus, and decreased visual acuity. As intracranial pressure decreases, patient headache and tinnitus symptoms can improve, but massive retinal apoptosis can lead to irreversible vision loss and even blindness due to prolonged edema of the retinal cells (17).

## 4. Major mechanisms by which cerebral venous diseases lead to impairment of visual function

Regarding the causes of visual impairment in CVT patients, the site of embolization was divided into two types: (1) Focal brain injury due to cortical vein thrombosis: focal cerebral edema (including vasogenic edema and cytotoxic edema), cerebral infarction, and intracerebral hemorrhage, which occurs in the visual center of the occipital cortex and will cause visual field defects; (2) Intracranial hypertension due to dural sinus thrombosis: Normally, the CSF drains through the arachnoid granulation located in the sagittal and transverse sinuses; however, dural sinus thrombosis can disrupt this buffering mechanism, resulting in increased venous pressure, decreased CSF absorption, and increased intracranial pressure. The patient's excessive intracranial pressure can involve the third, IV, and VI cranial nerves to cause diplopia and also directly causes optic nerve damage (2).

The mechanism by which CVSS causes visual impairment is mainly through optic nerve damage due to increased intracranial pressure. The pathogenesis of optic disc edema in raised intracranial pressure is a mechanical phenomenon. It is primarily due to a rise of raised cerebrospinal fluid pressure in the optic nerve sheath, which produces axoplasmic flow stasis in the optic nerve fibers in the surface nerve fiber layer and prelaminar region of the optic nerve head. Axoplasmic flow stasis then results in swelling of the nerve fibers, and consequently of the optic disc. Swelling of the nerve fibers and of the optic disc secondarily compresses the fine, low-pressure venules in that region, resulting in venous stasis and fluid leakage; that leads to the accumulation of extracellular fluid. Thus, optic disc edema in raised cerebrospinal fluid pressure is due to a combination of swollen nerve fibers and the accumulation of extracellular fluid (18). Visual function impairment often presents as transient blurring of visual objects, enlargement of physiological blind spots, visual field defects, and irreversible vision loss (18).

## 5. Treatments of visual impairment caused by intracranial hypertension

Apart from using systemic anticoagulants to attempt recanalization and drugs with carbonic anhydrase inhibitor activity to reduce the ICPs, surgical treatment for cerebral venous disease, including CVST and CVSS, also include (1) recanalization by local thrombolysis, stenting, or mechanical devices; (2) cerebrospinal fluid diversion procedures such as ventriculoperiotoneal shunting; and (3) specific treatment for conditions such as dural arteriovenous fistula (DAVF) occurring as a late complication (19–21). However, for visual impairment due to intracranial hypertension caused by CVT/CVSS, these methods do not provide rapid relief of optic nerve damage.

Patients with ocular symptoms and signs despite maximally tolerated medical treatment, ONSF is an effective and safe surgical option for the treatment of papilledema due to CVT/CVSS after medical treatment has failed (22). ONSF is a procedure that incises the optic nerve sheath in the intraorbital segment, thereby draining CSF into the orbital tissue to reduce intracranial pressure. The mechanism of ONSF is to provide a stoma to drain CSF or a fenestration to form a cyst to reduce pressure around the optic nerve (22). At present, the mechanism underlying the long-term onset of action is controversial and may be related to the closure of fistulas by fibroblasts in the subarachnoid space from the optic nerve sheath, scar formation, and a reduction in the distant conduction pressure (23). ONSF does not treat the source of the elevated ICP itself, but the procedure does diminish the associated visual sequelae (24).

Except for cerebral venous diseases, ONSF is widely applicated in papilledema due to IIH, cryptococcal meningitis, chronic inflammatory demyelinating polyneuropathy, intracranial mass or tumors, shunt malfunction (25–27). Although studies have reported that unilateral ONSF significantly decreases the grade of papilledema in both ipsilateral (operated) and contralateral (unoperated) eyes (28, 29), whether or not to operate on both eyes depends on the doctor and patient's condition. In addition, study suggests that secondary and tertiary ONSF is a viable management option for patients with progressive vision loss from IIH (30).

At present, there is no consensus on the timing of ONSF surgery. However, strict follow-up and timely ONSF are important to prevent irreversible visual impairment.

**Figure 1:**
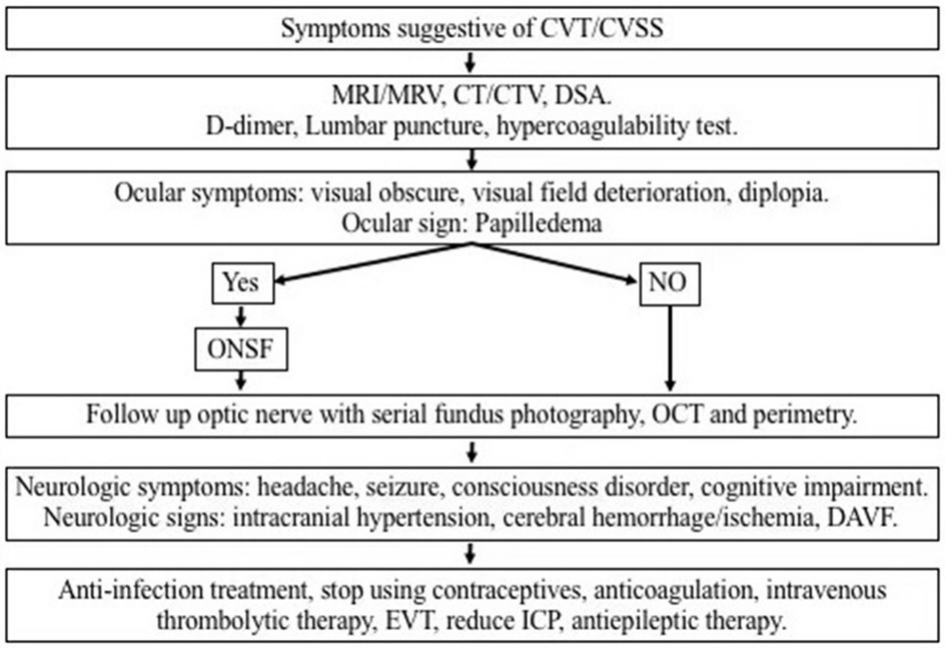
Schematic diagram for the proposed management algorithm of visual impairment caused by intracranial hypertension due to CVT/CVSS. CVT, cerebral venous thrombosis; CVSS, cerebral venous sinus stenosis; MRI, magnetic resonance imaging; MRV, magnetic resonance venography; CT, computed tomography; CTA, computed tomographic angiography; ONSF, optic nerve sheath fenestration; OCT, optical coherence tomography; DAVF, dural arteriovenous fistula; EVT, endovascular therapy; ICP, intracranial pressure.

Figure 1 provides a schematic diagram for the proposed management algorithm of new cases of CVT/CVSS.

## 6. History of ONSF treatment for intracranial hypertension secondary to cerebral venous diseases

ONSF was first reported and proposed by De Wecker in 1872 adapted in neuroretinitis. In 1964, Hayreh et al. successfully made an animal model of intracranial hypertension, implemented ONSF in this animal model and eliminated papilledema after surgery. In 1988, Brourman et al. believed that ONSF was the preferred surgical method to maintain or improve visual function in patients with IIH. In 1991, Sergott et al. first applied ONSF to the treatment of CVST and concluded that the prognosis was closely related to the intervention time. In 2005, Cunha et al. reported a case of chronic intracranial hypertension secondary to multiple cerebral venous thrombosis, after ONSF treatment, papilledema eliminated, but visual acuity continued to deteriorate. In the following years, several authors such as Nithyanandam, Murdock, and Moreau successively published original articles on the application of ONSF for cerebral venous diseases. And the development of history of ONSF as a treatment for intracranial hypertension is presented in Table 1.

**Table 1:** History of development of ONSF for intracranial hypertension.

**References**	**Disease/model**	**ONSF related description**
Moreau et al. (31)	Neuroretinitis	First report on ONSF.
Hayreh (32)	Animal model of intracranial hypertension	After ONSF, intracranial hypertensive papilledema was successfully eliminated.
Brourman et al. (33)	IIH (6 cases)	ONSF is the preferred way to improve vision in IIH patients.
Sergott et al. (34)	Sagittal sinus thrombosis (five cases)	With the first application of CVST, postoperative visual acuity has been improved, and the long-term prognosis is poor. It is suggested that the prognosis is closely related to the intervention time.
Acheson et al. (35)	Sagittal sinus and transverse sinus thrombosis (two cases)	50% of patients' visual acuity improved, visual field improved.
Banta and Farris (36)	IIH (86 cases)	97% of patients had stable or improved visual acuity and 88% had stable or improved visual field.
Cunha et al. (37)	Multiple venous thromboses in superior sagittal sinus, transverse sinus, and sigmoid sinus (one case)	Papilledema improved, vision deteriorated unceasingly.
Nithyanandam et al. (38)	Postpartum CVST (seven cases), other CVST (nine cases)	More than 80% of patients' visual acuity improved or stabilized.
Murdock et al. (22)	Factor V Leiden mutation, G20210A gene mutation, Factor XII deficiency, Decreased anticoagulant factor III and S protein, History of oral contraceptives CVST (one case)	Visual acuity and visual field improved; anticoagulation combined with ONSF is effective.
Moreau et al. (31)	CVST (seven cases)	ONSF has high security.
Elnahry et al. (39)	Antiphospholipid syndrome with CVST (one case)	Vision improved.
Bajin et al. (40)	IIH (56 cases)	The visual acuity and visual field of most of the operative and non-operative patients' eyes were improved, and the earlier the treatment, the better the effect.

## 7. Surgical approaches for ONSF

There are many surgical routes for ONSF, including the transconjunctival approach, transcranial route, and transnasal approach. According to a 2015 survey of ophthalmologists who performed ONSF (41), the three most commonly used surgical approaches were the medial conjunctival approach (59%), upper medial eyelid fold incision (31%), and lateral orbital incision (10%).

### 7.1. The medial conjunctival approach

**Figure 2:**
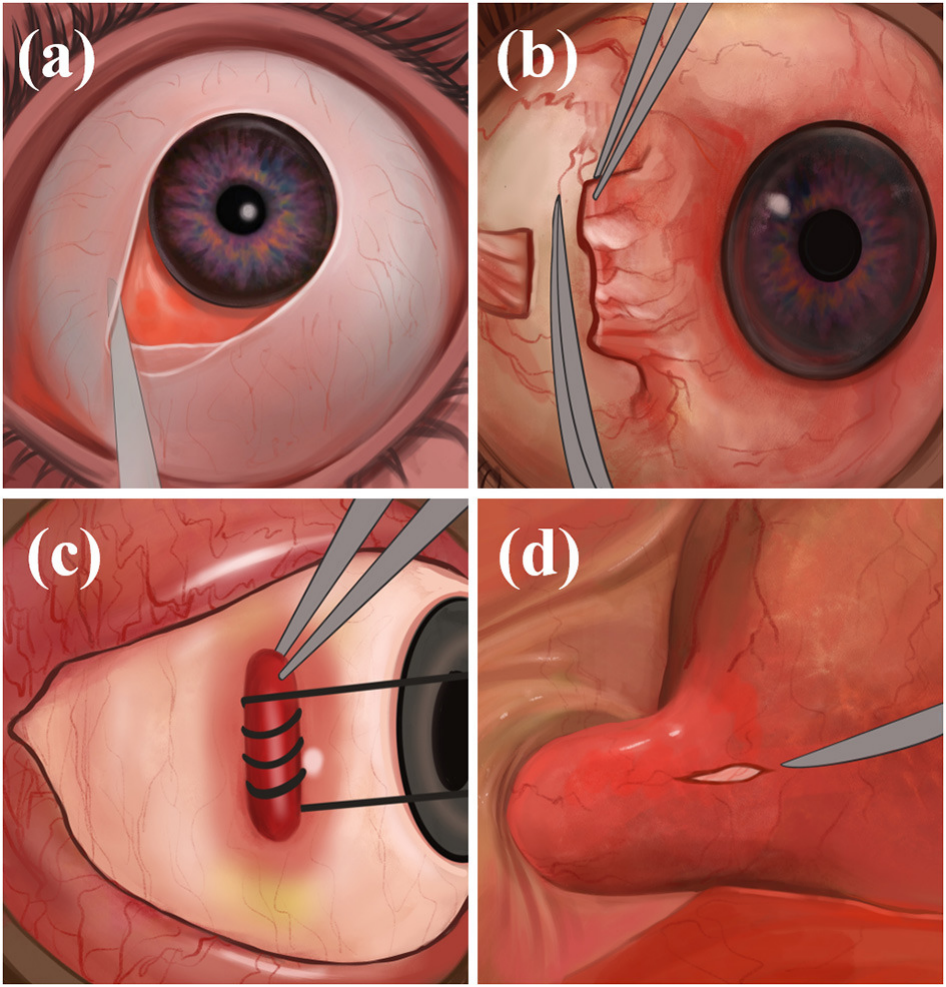
The medial conjunctival approach: **(a)** Incise the bulbar conjunctiva along the nasal limbus, bluntly separate the bulbar conjunctiva and fascial tissue; **(b)** expose and separate the medial rectus muscle; **(c)** immobilize the internal rectus muscle; **(d)** expose the optic nerve sheath and make an incision.

The physician makes an incision on the bulbar conjunctiva along the nasal limbus of the affected eye, bluntly separates the bulbar conjunctiva and fascial tissue, and exposes and separates the medial rectus muscle. The physician makes a traction suture and pulls the eyeball against the temporal side, deflects, pushes the periocular and retro-orbital fat against the nasal side, and fully exposes the optic nerve sheath and posterior ciliary vessels and nerves. Finally, the optic nerve sheath is incised to drain the CSF, the rectus muscle is repositioned, and the conjunctiva is sutured (Figure 2). Through the medial conjunctival approach, the ONSF surgical approach is less surgically invasive than other approaches, leaving no scar after surgery and shorter recovery time. However, because of the damage to the medial rectus muscle and ciliary ganglion as well as blood vessels, it has the potential to cause postoperative short-term diplopia, pupillary paralysis, elevated intraocular pressure (IOP), and retinal artery occlusion (42).

### 7.2. Transepithelial medial eyelid approach

**Figure 3:**
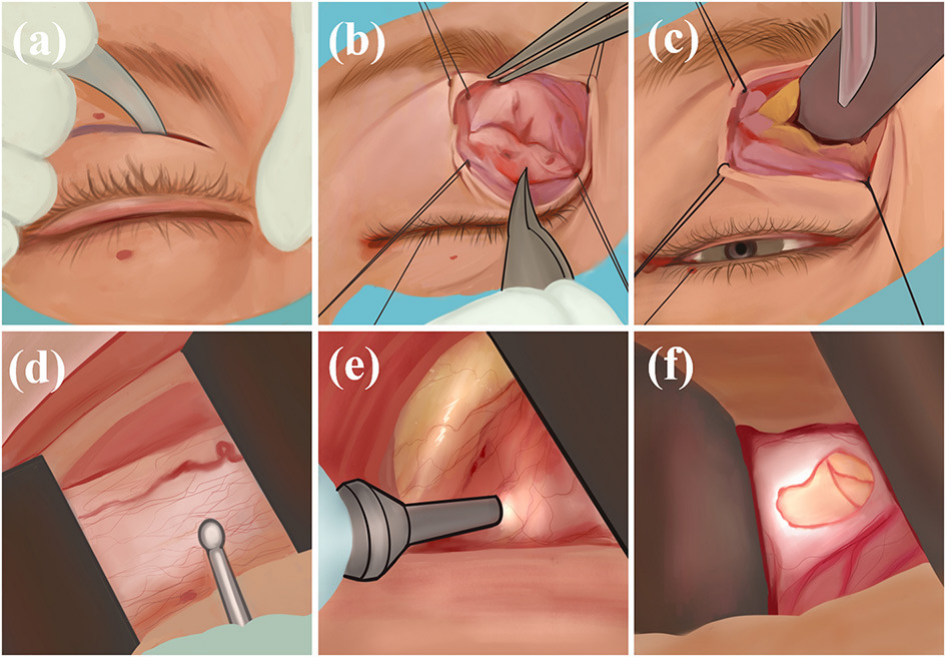
Transepithelial medial eyelid approach: **(a)** Incise the skin; **(b)** incise orbicularis oculi muscle; **(c)** bluntly separate the medial fat in the upper eyelid; **(d)** expose the optic nerve sheath; **(e, f)** incise and drain.

The physician makes an incision in the skin and orbicularis oculi muscle along the medial upper palpebral fold, uses sutures to secure the margins, bluntly separates the medial fat in the upper eyelid, exposes the optic nerve sheath, and incises and drains the CSF (Figure 3). The transepithelial medial eyelid approach saves time and does not result in injury to the extraocular muscles, far from the ciliary ganglion, reducing the probability of diplopia and pupillary paralysis complications; however, its disadvantage is the small surgical field and that there may be scar postoperatively (43).

### 7.3. Transcanthal incision approach

**Figure 4:**
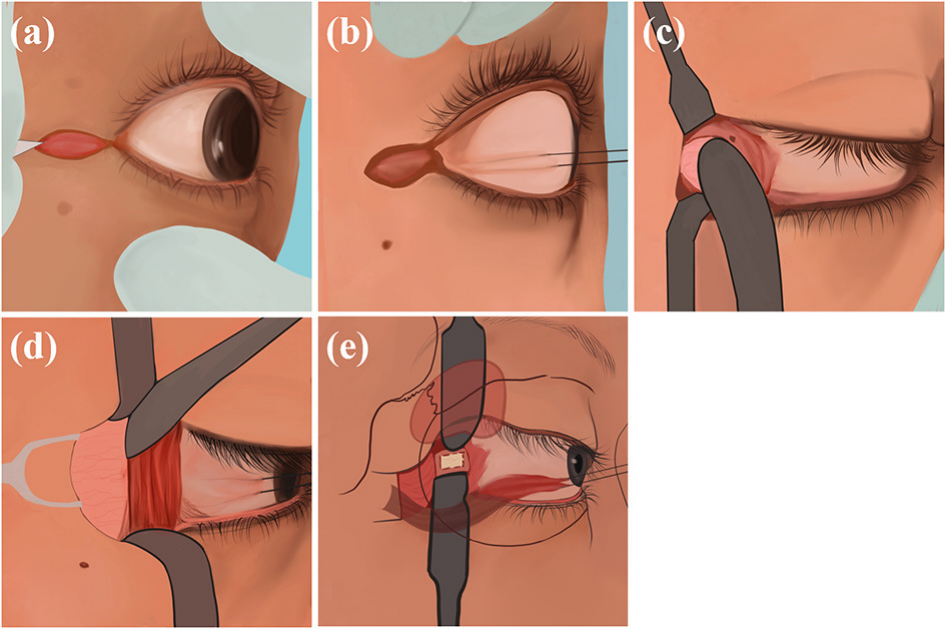
Transcanthal incision approach: **(a)** Incise a 1 cm lateral canthal incision; **(b)** use sutures to secure the lateral rectus muscle; **(c)** separate the overlying periosteum superiorly and inferiorly; **(d)** expose the eyeball, lift the deep lacrimal gland; **(e)** expose the optic nerve sheath and make an incision.

The physician creates a 1-cm lateral canthal incision, uses sutures to secure the lateral rectus muscle by traction under the lateral rectus muscle, and separates the overlying periosteum superiorly and inferiorly at the lateral margin. During the operation, the physician aims to expose the patient's near orbital rim eyeball, lift the deep lacrimal gland, expose the optic nerve sheath, and make an incision (Figure 4). Using this technique, a large enough surgical field can be obtained and the success rate of safe surgical manipulation of the optic nerve is guaranteed. At the same time, physicians there is no damage to the extraocular muscles, reducing the probability of diplopia; however, there are risks of damage to the lacrimal and ciliary ganglion causing lacrimal gland dysfunction and pupillary paralysis to a certain extent (44).

### 7.4. Nasoendoscopic transsphenoidal route

**Figure 5:**
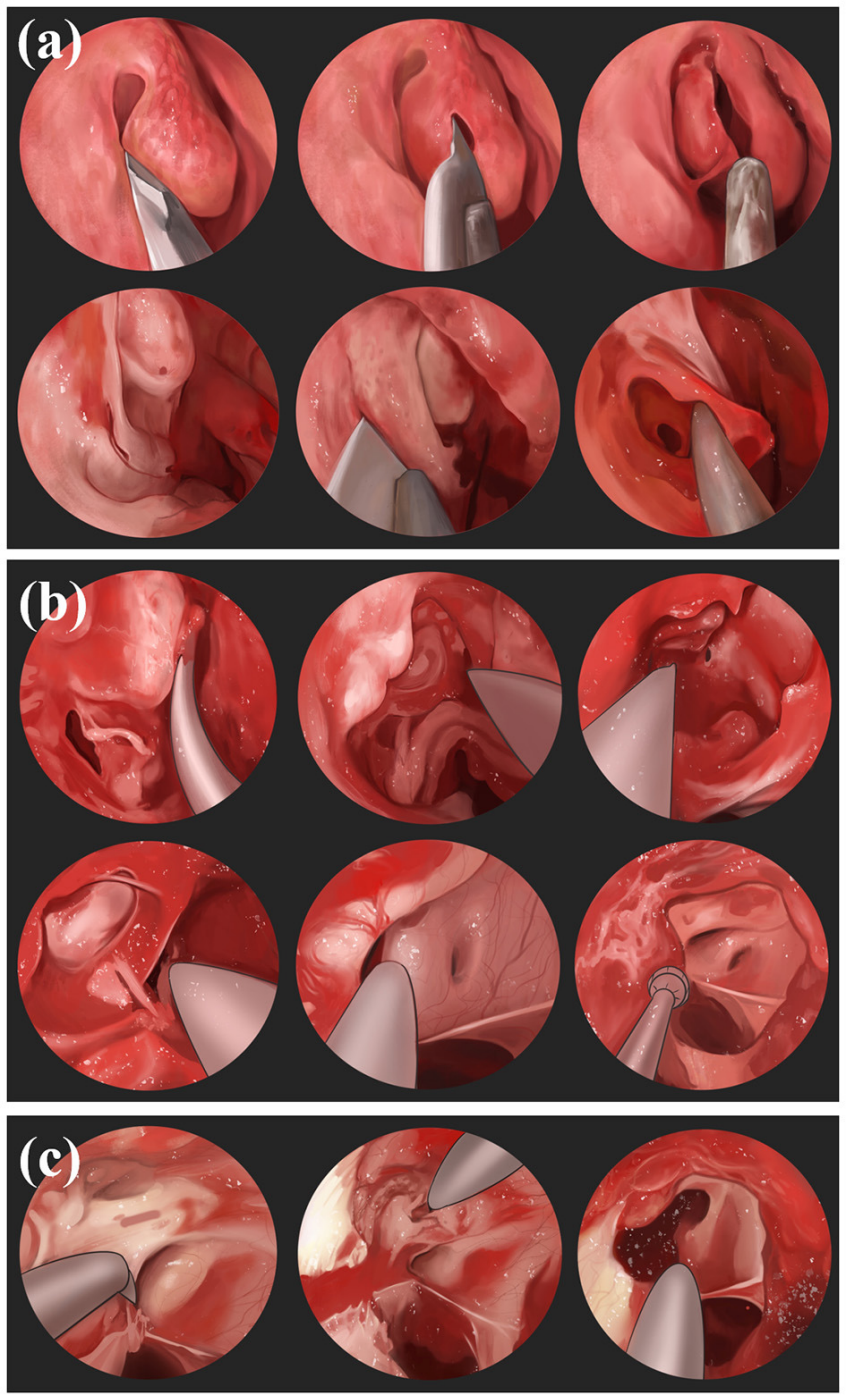
Nasoendoscopic transsphenoidal route: **(a)** Use nasendoscopy to pass through the nostril, resects part of the middle turbinatet, exposes the uncinate process and ethmoid bullae of maxillary sinus, and incises the lower 1/3 of the maxillary sinus to expose the maxillary sinus. **(b)** Resect the ethmoid and sphenoid sinuses, reveal the sphenoid plateau and sellar base, identify the optic nerve carotid crypts as well as the optic nerve bulging. **(c)** Chisel the medial and superior wall of optic nerve canal and make an incision from medial to lateral.

The physician uses nasendoscopy to pass through the nostril, resect part of the middle turbinate of the patient, expose the uncinate process and ethmoid bullae of the patients' maxillary sinus, and incise the lower 1/3 of the maxillary sinus to expose and enlarge the maxillary sinus to prevent mucous cyst formation. Then, the physician resects the ethmoid and sphenoid sinuses, revealing the patients' sphenoid plateau and sellar base, identifies the optic nerve carotid crypts as well as the optic nerve bulging, and then chisels the medial and superior wall of optic nerve canal and makes an incision from medial to lateral (Figure 5). The advantage of this procedure is the short operation time, which allows simultaneous bilateral optic nerve canal decompression. The disadvantage is the risk of complications such as CSF leakage, infection, and epistaxis (45).

### 7.5. Transcranial route

**Figure 6:**
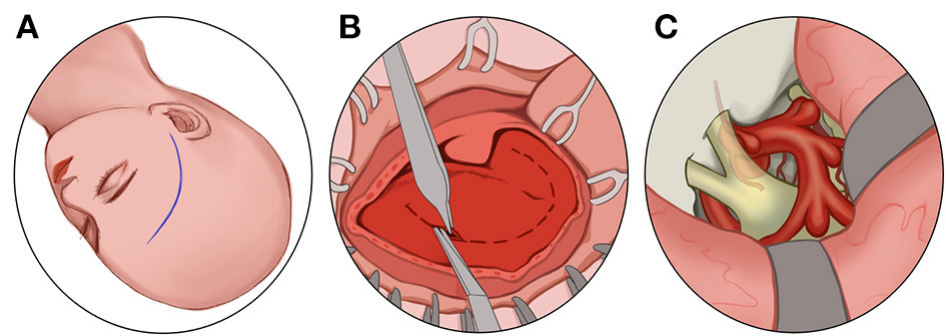
Transcranial route: **(A)** Make a frontotemporal pterygium craniotomy *via* Dolenc route. **(B)** cut the scalp and temporalis muscle. **(C)** Grind off the anterior bed and the sphenoid bone and decompress the optic nerve at 270 degrees from superior, medial, and inferolateral.

The physician adopts a frontotemporal pterygium craniotomy *via* Dolenc. The patient takes the supine position, and the head is rotated to the contralateral side by 30 degrees and is fixed using a head frame so that the supraorbital rim and zygomatic arch junction are at the highest point. The physician cuts the patients' scalp and temporalis muscle hierarchically, generating a free bone flap. The physician resects the sphenoid crest and part of the orbital roof of the patient under the microscope, cuts the dural sheath transversely through the supraorbital fissure, and uses an ultrasonic aspirator to separate the two-layered dural gaps in the lateral wall of the patient's cavernous sinus. The physician grinds off the patients' anterior bed process and continues with the abrasive decompression along the optic column to the sphenoid bone to decompress the patients' optic nerve at 270 degrees from superior, medial, and inferolateral. The physician then incises the patients' optic nerve sheath, which, together with the drilled bone, forms a channel for CSF outflow, and the fistula in this way communicates with the overlying muscles and the frontal and temporal lobes for systemic absorption (Figure 6). The advantage of such manipulation is that it is performed transorally without the risk of damaging the patients'vessels, resulting in a low incidence of diplopia. The disadvantages are the risk of infection and subdural cyst (46, 47).

### 7.6. Superonasal transconjunctival

Melson et al. (48) use an incision made through the conjunctiva in the superonasal fornix, excise a small strip of the underlying Tenon capsule, secure the superior and medial rectus muscles with separate 4-0 silk traction suture, insert The Farris-Tang retractor through the conjunctival incision to retract the surrounding structures nasally, expose the optic nerve sheath and create a fenestration along the superonasal aspect of the optic nerve sheath with intraocular scissors. The advantage of this procedure is the lack of muscle disinsertion which reduces postoperative diplopia rates as well as increases the efficiency and shortens operative time. Any intraoperative or perioperative complications have not be identified.

### 7.7. Emerging technologies

Kozub et al. (49) discovered that doctors could expose the optic nerve through an endoscope and then use infrared lasers for sheath cautery. Mitros et al. (50) developed a three-arm robot called the Concentric Tube Robot (CTR). The robot is equipped with a video camera, drilling needle, and micro holder, which can be self-regulated according to the curvature of the patient's ocular surface, entering from the front of the patient's eye, reaching the orbit and optic nerve, and completes a precise sheath incision operation.

## 8. Prognostic assessment for the ONSF treatment of papilledema

Previous studies have suggested that various surgical approaches and ways of cutting the optic nerve sheath (remove a window or fenestrate with slits) can achieve the purpose of decompression of the optic nerve sheath, with few complications (41, 43, 44, 51–56). The rare complications of ONSF surgery include transient vision loss, choroidal infarction, transient third and sixth nerve paresis (57–59). Regarding the evaluation of whether the procedure is effective, the current mainstay is clarification by comparing retinal nerve fiber layer thickness with optical coherence tomography, b-super-sheath diameter, optometry for best-corrected visual acuity, and Humphery perimetry (60, 61). Factors that may affect the surgical outcomes include inadequate exposure to the optic nerve, insufficient depth of optic nerve sheath incision, poor postoperative fibroblast proliferation or premature excessive proliferation of scar tissue, and, most importantly, the timing of the surgery for ONSF. Obi et al. (62) suggested that ONSF should be implemented aggressively and early to avoid a progressive decline in visual function in patients with IIH. Robinson et al. (63) suggested that for IIH patients with intracranial pressure higher than 50 cmH_2_O, the postoperative visual function improvements such as visual acuity and visual field were largely inferior to those with intracranial pressure lower than 50 cmH_2_O, and that the probability of poor visual outcome was also higher.

## 9. Prospects

With the improvement of specific details of the ONSF procedure and surgical instruments, complications of ONSF have reduced and its safety has been significantly improved. Although the number of clinically investigated cases in the literature remains low and there is a lack of controlled studies, the available studies are sufficient to suggest that ONSF can be used to prevent, or even reverse, visual impairment in patients with cerebral venous diseases, especially in patients who fail to respond to other treatments and still have progressive visual loss. Operation time is essential to save the patient's visual function; the earlier the operation time, the less damaged the optic nerve, and the greater the likelihood of recovery of visual function. Therefore, scholars are actively studying the optimal surgical timing of patients. In addition, it is necessary to develop a screening model for the patient's suitability for surgery. Furthermore, while pursuing improvements in visual function, we should actively search for a cause, cooperate with other regimens, and allow patients to receive timely and appropriately effective treatment.

## Data availability statement

The original contributions presented in the study are included in the article/supplementary material, further inquiries can be directed to the corresponding author.

## Author contributions

All authors listed have made a substantial, direct, and intellectual contribution to the work and approved it for publication.
